# Immunomodulating Activity and Therapeutic Effects of Short Chain Fatty Acids and Tryptophan Post-biotics in Inflammatory Bowel Disease

**DOI:** 10.3389/fimmu.2019.02754

**Published:** 2019-11-22

**Authors:** Edda Russo, Francesco Giudici, Camilla Fiorindi, Ferdinando Ficari, Stefano Scaringi, Amedeo Amedei

**Affiliations:** ^1^Department of Clinical and Experimental Medicine, University of Florence, Florence, Italy; ^2^Department of Health Professions, Dietary Production Line and Nutrition, Azienda Ospedaliera Universitaria Careggi, Florence, Italy; ^3^SOD of Interdisciplinary Internal Medicine, Azienda Ospedaliera Universitaria Careggi, Florence, Italy

**Keywords:** gut microbiota, infammatory bowel disease, immunonutrition, post-biotics, SCFAs, tryptophan, metabolites

## Abstract

Crohn's disease (CD) and Ulcerative colitis (UC) are grouped as Inflammatory Bowel Diseases (IBD). The IBD is associated to a multifaceted interplay between immunologic, microbial, genetic, and environmental factors. Nowadays, the gut microbiota (GM) dysbiosis has been indicated as a cause in the IBD development, affecting the impaired cross-talk between GM and immune cells. Moreover, recent studies have uncovered a crucial role for bacterial post-biotics (metabolites) in the orchestration of the host immune response, as they could be messengers between the GM and the immune system. In addition, transgenic mouse models showed that SCFAs (Short Chain Fatty Acids) and Tryptophan (Trp) post-biotics play important immunomodulatory effects, regulating both innate and adaptive immune cell generation, their function and trafficking. Here, we present an overview on the main microbial post-biotics and their effects on the gut mucosa with specific emphasis on their relevance for IBD. Finally, we discuss the therapeutic potential of SCFA and Trp post-biotics on IBD through approaches based on the “immunonutrition,” defined as a modulation of the immune system provided by specific interventions that modify dietary nutrients.

## Introduction

Inflammatory bowel disease (IBD) is a multifactorial chronic inflammatory disorder of the intestine that can be divided into two principal clinical conditions: Crohn's disease (CD), which mainly concerns the colon and small intestine, and Ulcerative colitis (UC) that is restricted to the colon. The two IBD subtypes are characterized by chronic inflammation of the gastrointestinal tract with recurrent cycles of remission and relapse. With over 1 million inhabitants estimated to be affected in the US and 2.5 million in Europe, IBD represents a widespread condition (1). At present, the complete IBD etiology and pathogenesis remain to be clarified. Meanwhile the incidence steadily increased worldwide (2). Although UC and CD appear dissimilar in their clinical presentation, researchers assume that common risk factors could be involved in their pathogenesis (3). The association with the host genetic susceptibility (NOD2, TLR4, CARD9, ATG16L1, IL23R polymorphisms), immunological abnormalities, the key role of gut microbiota (GM) and its produced metabolites, and other environmental factors, have been recently investigated (4).

Impairments in IBD immunological regulation have been described at different levels, involving epithelial damage, inflammatory cells infiltrating into the *lamina propria* and failure of immune regulation in controlling the inflammatory response. Regarding the lymphocyte immune response, CD was described as a Th (T helper)1/Th17 condition, whereas UC is related with an exaggerated Th2 mediated response (5).

Nowadays, it is recognized the physiological importance of a mutual interplay between host immune response and GM (6). A dysbiotic microbiota (a variation from the “healthy” GM structure and function) has been involved in several diseases including type 2 diabetes, colon cancer, and obesity (6–9). The pattern of dysbiosis has an impacting role also in the IBD pathogenesis and prognosis (10). Of note, the inflammation in IBD is generally found in the distal ileum or colon that are the intestinal sites where the microbial abundance is higher. In addition, a significant difference in the GM of healthy individuals and IBD patients in terms of diversity and load has been confirmed (11, 12). An overall reduction in the total number of species and a decrease in diversity of the GM has been associated to IBD. In particular, different human studies show a reduced abundance of commensal bacteria, particularly in *Bacteroides* and *Firmicutes*, and a relative increase of bacterial species belonging to *Enterobacteriaceae* (13–17). Other human studies have confirmed a clear reduction in *Firmicutes* (especially *Clostridium* groups) diversity and an increase in *Proteobacteria* (18, 19) alongside a decrease of many other beneficial bacterial species from the genera *Bacteriodes, Lactobacillus*, and *Eubacterium* (20). Moreover, an increased abundance of *Ruminococcus gnavus* and a decrease in *Faecalibacterium prausnitzii, Bifidobacterium adolescentis, Dialister invisus*, and an unknown member of *Clostridium* cluster XIVa has been demonstrated for CD (21). Interestingly, *Faecalibacterium* showed a protective role against inflammation of the colon mucosa (22). Curiously, increased levels of *Faecalibacterium prausnitzii* were found in UC (23). Besides *F. prausnitzii*, also *A. muciniphila* has been associated with dysbiosis in IBD. The study of Lopez-Siles et al. showed a slight underrepresentation of *A. muciniphila* in the colonic mucosa of CD patients, regardless of disease activity status; in particular, early onset CD was characterized by a lack of *A. muciniphila* (24). In colitis mouse models, *A. muciniphila* treatment ameliorated mucosal inflammation either via microbe-host interactions, which protect the gut barrier function reducing the levels of inflammatory cytokines, or by improving the microbial community, suggesting that *A. muciniphila* may be a potential probiotic agent for ameliorating colitis (25). Besides bacteria, recent studies focused on viruses, fungi and archaea in the IBD scenario. Microbiota comprises both prokaryotic and eukaryotic viruses, that together compose the gut virome. A recent work of Ungaro et al. profiled the gut eukaryotic virome in young treatment-naïve patients with early-diagnosed IBD and identified the eukaryotic viral communities that might be involved in IBD onset. In particular, *Herpesviridae* family was highly abundant in all the analyzed eukaryotic gut viromes, although not differentially enriched among the groups. *Hepeviridae*-derived proteins may have an impact on host immunity, eventually triggering intestinal inflammation. Conversely, other viral families, such as *Polydnaviridae* and *Tymoviridae* in UC, and *Virgaviridae* in CD, were less enriched in IBD patients and negatively correlate with the presence of other viruses; these condition might be somehow considered protective in the human host (26). In addition, a role of the mycobiota (the fungal component) in IBD, is also indicated by both descriptive data in humans and mechanistic data in mice. Intestinal or distal inflammation occurring in diseases associated with an increased intestinal permeability might be related to β-glucan translocation (27). Moreover, recent evidence has shown that the variable prevalence of Archaea methanogens (an ancient domain of single-celled organisms) could have certain effects on IBD. A recent human study has shown a reverse association between *Methanobrevibacter smithii* bacterial load and susceptibility to IBD, and this association could be extended to IBD patients in remission (28). Finally, IBD patients harbor only 25% (fewer) of the mucosal microbial genes of healthy individuals (29) and the altered GM profile has been documented in fecal and mucosa samples (30, 31). Moreover, a recently published human study demonstrates that the GM and the molecular functional profile in terms of transcriptome and metabolome, and the host immune factors, are crucial to IBD (32).

Although the cited spectrum of published research widely recognizes the dysbiosis, which occurs in IBD patients, the causal role of dysbiosis has not yet been established. Nowadays, host-microbiome relation in IBD has just begun to be uncovered and so other types of mechanisms could be involved, such as aberrant cell-to-cell interactions and the production, conversion, and sensing of bacterial bioactive small molecules, named “post-biotics.” Post-biotics have recently been proposed as “non-viable” bacterial products or metabolic byproducts (metabolites) from probiotic microorganisms that promote biological activity in the host (33).

The microbiome-modulated post-biotics (MMPBs) may influence the host cellular pathways involving proliferation, differentiation, migration and cellular death. In addition, MMPBs could exert effects on maturation/function of mucosal and systemic immunity (34). The role investigation of bacterial post-biotics as messengers between the GM and the immune system could have a great impact in elucidating impaired host–microbial interactions in IBD. Further comprehension on how the GM metabolism can shape the host immune system (immunomodulation) might improve the translation toward clinical applications.

In this review, we present the main immune functions of relevant MMPBs as Short Chain Fatty Acids (SCFA) and Tryptophan (Trp) catabolites (35) along with recent observations that could link metabolite misbalances to IBD. Furthermore, as many IBD patients do not respond adequately to the therapeutic treatments, or they show acute side effects, future therapeutic approaches are required: we discuss post-biotics supplementation as a therapeutic strategy that could modulate the host immune response through a recent approach named “Immunonutrition.”

## Relevant Microbiota Post-Biotics in IBD Pathogenesis

The commensal gut bacteria produce an extremely various repertoire of metabolites, including SCFAs, tryptophan catabolites, essential vitamins (e.g., group B and K vitamins), phenolic acids and bile acids. The MMPBs play distinct bioactive functions for the host cells (36), determining thus multiple pathophysiological effects. A GM comparative evaluation of healthy individuals and IBD patients demonstrated that the speciation of the GM (genus-level clades) from healthy vs. IBD differed by 2%, but the metabolic profiles of the respective GM differed by 12% (37). This study demonstrated that perturbations in bacterial composition, although modest, are associated with major perturbations of GI microbiome function suggesting that the bacterial production of metabolites might perturb the host in other manners that are significant for IBD pathogenesis. Future studies focused on metabolomic characterization will be needed to additionally define the consequences of the IBD-associated microbiome dysfunction on the host and the specific mechanisms by which they are carried out or regulated by the microbiota.

Several commensal bacteria, including *Enterococcus* spp, *Enterobacteriaceae*, and *Lactobacillus* spp., can influence the general MMPBs amount, for example by requiring specific amino acids as nitrogen sources, or by producing secondary metabolites (38). In the intestinal tract, the interface between the host and its GM is composed of a monolayer of epithelial cells that allow the access and interaction with the metabolites produced by the GM.

SCFAs are the most studied microbial metabolites in IBD. They are secondary metabolites produced through the fermentation of dietary substrates, such as proteins, peptides, resistant starches, and undigested fibers by the GM. So far, the dysbiotic condition in IBD patients has been associated with impaired SCFAs-fermentative pathways, which reflects a decreased number of SCFAs-producing bacteria and a lower amount of fecal SCFAs (39). Moreover, SCFAs production has been associated with a reduced IBD risk (40). SCFAs are a group of fatty acids with less than six carbons (including acetic, formic, propionic, butyric, and valeric acid) whose production is influenced by several factors, such as host nutrition, and GM diversity in terms of presence/absence and concentration of specific commensal bacteria (41). Propionate and acetate are produced mainly by *Bacteroidetes* members, while butyrate is mostly produced by the *Firmicutes* phylum. Within the eukaryotic host, SCFAs can be used as an energy source by the colonocytes, or they can be conveyed to blood circulation and other tissues. Interestingly, the main SCFAs (i.e., butyrate, acetate, and propionate) possess different production ratios and physiological activities, and the final composition given by these acids can change throughout the different sites of the whole gut. In detail, propionate and acetate are observed in both large and small intestines, while a greater concentration of butyrate was found in the cecum and colon (42). As for the SCFAs synthesis, propionate can be produced through the lactate pathway by *Firmicutes*, and/or succinate pathway by *Bacteroidetes* phylum (43). The recent discovery about the capability of SCFAs to bind receptors, such as GPR41, GPR43, and GPR109a (usually expressed on a large number of cell types) allowed clarifying the regulatory activity of SCFAs in IBD. Beyond the use of SCFAs as an energy source by intestinal epithelial cells (IECs), the SCFAs exhibit modulating effects on immune system cells (e.g., T cells, especially Tregs, neutrophils, and macrophages). In fact, the SCFAs can affect cytokine production and migration, cytolytic activity, and epigenetic modulation.

In addition to altered SCFAs concentrations in IBD patients, metabolomic analyses showed a decreased serum level of Trp and Trp metabolites (44). The bacterial metabolism of Trp may involve different pathways being substrate of both the gut mucosa and GM enzymes (45). Trp derives from dietary substrates and it is absorbed by SLC6A19/B0AT1 (a sodium-dependent neutral amino acid transporter) (46). Trp is a precursor for several MMPBs and can exert different functions on the host, such as immune homeostasis, but also with inflammatory response. The Trp availability is essential for the protein synthesis, production of indole and nicotinamide derivatives *via* kynurenine, as well as for the serotonin synthesis (47).

## IBD and the MMPBs Immunomodulating Activity

**Figure 1:**
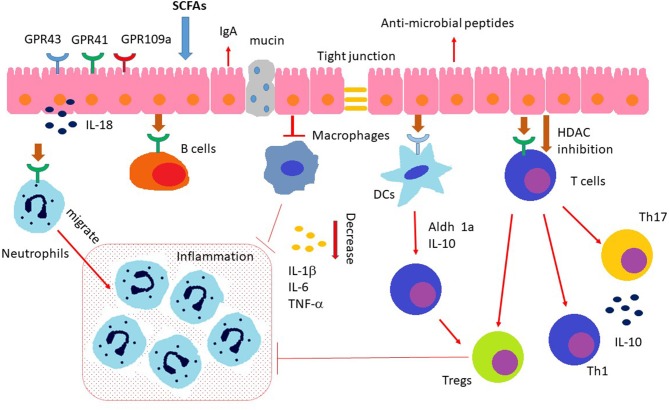
Immunomodulating effects of SCFAs. SCFAs regulate the gut barrier integrity by promoting intestinal epithelial cell secretion of IL-18, mucin, antimicrobial peptides, and upregulating the expression of tight junctions. In addition, SCFAs (a) induce neutrophils chemotaxis to inflammatory sites and enhance their ability of phagocytosis; (b) regulate the T cell functions through both GPCR pathway and inhibition of HDAC (histone deacetylases); (c) regulate the differentiation and functionality of Th17, Th1, and Tregs; (d) inhibit intestinal macrophage generation of proinflamamtory cytokines through inhibition of HDAC; (e) induce intestinal IgA production of B cells.

Recent studies reveal that the host immune system can “sense” the MMPBs repertoire, and this recognition can induce (by several mechanisms) immunomodulation, causing IBD and the consequent inflammation of the gastrointestinal tract (48). Given its vast superficial area and continuous exposure to the host microenvironment, the gut epithelial barrier is receptive to any potential damage induced by pathogens and toxins. The interplay immune–microbiota is so complex that various animal models for colitis need to explore the different possibilities of inducing chronic inflammation in genetically predisposed animals. Mucosal cellular subsets, such as IECs, DCs, macrophages, T cells, and innate lymphoid cells (ILCs), can express sensing platforms, regulating the mutualistic cross-talk between GM and the host. Furthermore, GM metabolites can move to other host organs, such as the central nervous system, where they can regulate the immune responses (9, 49). As previously reported, the dysbiotic condition in IBD may affect the impaired interplay between GM and immune cells also involving aberrant signaling through immunomodulatory metabolites. Actually, it is still under debate whether a dysbiotic microbiota in IBD patients has a primary pathogenetic role or is secondary to the inflammatory and antimicrobial responses elicited during the disease course (50). Among MMPBs, SCFAs might play crucial roles in each phase of the inflammatory process, regulating the function of almost every type of immune cells (Figure 1). In particular, butyrate has been demonstrated to have a complex regulatory role, exerting for example an anti-inflammatory effect on both immune cells and IECs at the gut level (51). Notably, SCFAs inhibit stimuli-induced expression of adhesion molecules, chemokine production, and consequently they suppress monocyte/macrophage and neutrophil recruitment, suggesting a potential anti-inflammatory role *in vivo*. In addition, several studies with IBD mouse models revealed a protective role of SCFAs (48). However, there is also some evidence that suggests a pro-inflammatory action of SCFAs. This discrepancy may be partially explained by the presence of microbes causing infections in anaerobic sites where, by the following loss of intestinal epithelial integrity, there is a high concentration of SCFAs *in loco* that may lead toward neutrophil accumulation and rise of inflammatory processes (52).

SCFAs-mediated immunomodulation could be regulated through different specific mechanisms: (I) activation of GPCRs, (II) stimulation of histone acetyltransferase, (III) inhibition of histone deacetylase (HDAC), and (IV) stabilization of hypoxia-inducible factors (53–56).

SCFAs are fundamental in preserving mucosal immunity since they improve the barrier activity of IECs (composed by mucus-secreting goblet cells, absorptive enterocytes, hormone-producing enteroendocrine cells, lectins-secreting Paneth cells, and antimicrobial peptides) that act on tight junction permeability (57). *In vitro* model of human cell showed that he gut epithelial goblet cells are able to promote the transcription of mucin genes in response to SCFAs stimuli (58). The IL-18 is involved in antimicrobial peptides synthesis, mucin production and in the control of the GM composition, has been shown to prevent a colitogenic phenotype in mouse (59). During the innate immune responses at mucosal sites, the TLRs of the different cells (in DCs, IECs, and neutrophils) might recognize the MMPBs. A mouse study proved that propionate and butyrate could inhibit maturation of DCs that represent a bridge between the innate and adaptive immune system (60). TLRs are strategic innate immune receptors able to detect pathogen-associated molecular patterns (PAMPs), which represent peculiar pathogenic “molecular signature.” After the PAMPs stimulation, the TLRs can initiate the inflammatory responses and erase the pathogenic invaders. A study showed that SCFAs impact on pro-inflammatory cytokines production (e.g., IL-6, IL-1β, IL-8, and TNF-α) in human IECs by increasing NF-κB stimulation in TLR ligand-responses (61). Notably, the impact of SCFAs on TNF-α and IL8 production provides another mechanism by with MMPBs would be expected to influence gut health. In particular, TNF-α and IL8 are also involved in maintaining epithelial homeostasis by influencing epithelial to mesenchymal transition (EMT) and the inverse process (MET) (62). This two process (EMT and MET) represents cellular trans-differentiation programmes by which epithelial cells acquire mesenchymal features and *viceversa* (63). Specifically in the context of intestinal disease, increasing evidence has supported a role for EMT in the pathogenesis of IBD-associated intestinal fibrosis (64, 65).

MoreoverSCFAs can modulate the activities of mouse DCs, which can produce cytokines and interact with T cells. The exposition of DCs to butyrate has been shown to increase naïve T-cells differentiation into Tregs, inhibiting the transformation of the same into IFN-γ-producing T cells (66). Butyrate can also modulate the macrophages' activity of colon lamina propria in mouse, inhibiting the transcription of proinflammatory molecules (e.g., Nos2, Il6, and Il12), providing a status of tolerance toward the GM (67).

Furthermore, the chemotaxis of neutrophils is induced by inflammatory mediators (e.g., IL-17, TNF-α, or chemokines) and the SCFAs might provoke the movement of neutrophils to inflammatory sites, increasing also their phagocytic activity. In detail, SCFAs induce the neutrophils' chemotaxis by activating GPR43 (52), in rats, modulating both the phagocytic activity and the production of reactive oxygen species (ROS) (68).

Regarding adaptive immune responses, SCFAs could also exert immunomodulating activity in T and B cells. SCFAs regulate the T cell differentiation that can be mediated with two types of processes: by indirect regulation of DCs (as previously reported) and through a direct effect on T cells. Finally, the induction of Th17, Th1, and Tregs is also modulated by SCFAs in different cytokine milieu (69).

Compared to healthy controls, IBD patients presented an augmented concentration of Tregs in gut and especially in inflammatory lesions (70). In detail, butyrate has been shown to induce epigenetic modifications, upregulating the histone H3 acetylation of Foxp3 and inducing the Tregs' differentiation, acting as an anti-inflammatory mediator (71). Butyrate might inhibit several zinc-dependent HDACs, leading to the hyperacetylation of histones. Consequently, the nuclear chromatin results in an open structure, thus making DNA accessible to genes transcription. Butyrate can inhibit (NF-kB) activation, containing the inflammatory response and reducing the production of proinflammatory molecules (72). Moreover, the nuclear peroxisome proliferator activated receptor (PPAR)γ, which could be induced by butyrate, is exerting an anti-inflammatory activity through NF-kB antagonism in murine cell culture (73). Products operating as HDAC inhibitors may be an efficient treatment for IBD and other pro-inflammatory cytokine-related diseases.

Conversely, different cell observations showed that butyrate can induce other epigenetic modifications, as hyperacetylation of non-histone proteins (74), histone methylation (75), selective inhibition of histone phosphorylation (76), and DNA methylation (77). In addition, HDAC inhibitory activity of butyrate stimulates gene expression alterations also in mouse DCs, including the suppression of IL-6, IL-12, and Relb, which influence the polarization of Tregs (78). In addition, *in vitro* studies demonstrated that the SCFAs could also influence human leukocyte function through inhibition of HDAC activity leading to NF-kB inactivation and suppression of proinflammatory cytokines and nitric oxide (79).

Most favorable immunomodulating roles of SCFAs in the gastrointestinal tract are mediated by the direct activation of its GPCR receptor (80). GPR109A acts also as receptor for bacterial-derived nicotinic acid (NA). On macrophages, IECs and DCs (isolated from mice), both the butyrate and NA could link GPR109A leading to the production of respectively IL-18 from IECs, and IL-10 from macrophages and DCs, which further induce the suppression of inflammation as IL-10 might promote the Tregs differentiation (81).

It has also been shown that in B cells, the SCFAs might favor the IgA secretion in mice (82). Plasma B cell differentiation could be promoted also by SCFAs, as they could modulate the gene expression that is necessary for antibody generation in a SCFAs receptor-independent manner (83). On the contrary, acetate could induce IgA production with GPR43-dependent way (84). GPR43 could have a role in the modulation of gut inflammation, which may be related cytokine production and neutrophil recruitment. In colitis' mouse models, lacking GPR43, the mice develop exacerbated inflammation due to a greater generation of inflammatory effectors and an augmented enrolment of immune cells (85).

Elucidating the multifaceted molecular processes underlying the anti-inflammatory activity of SCFAs, it has been challenging because they could act together with different signaling compound. As the effects of SCFAs are sometimes combinatorial, diverse, and indirect, future investigations will have to explain their therapeutic prospective in clinical aspects.

**Figure 2:**
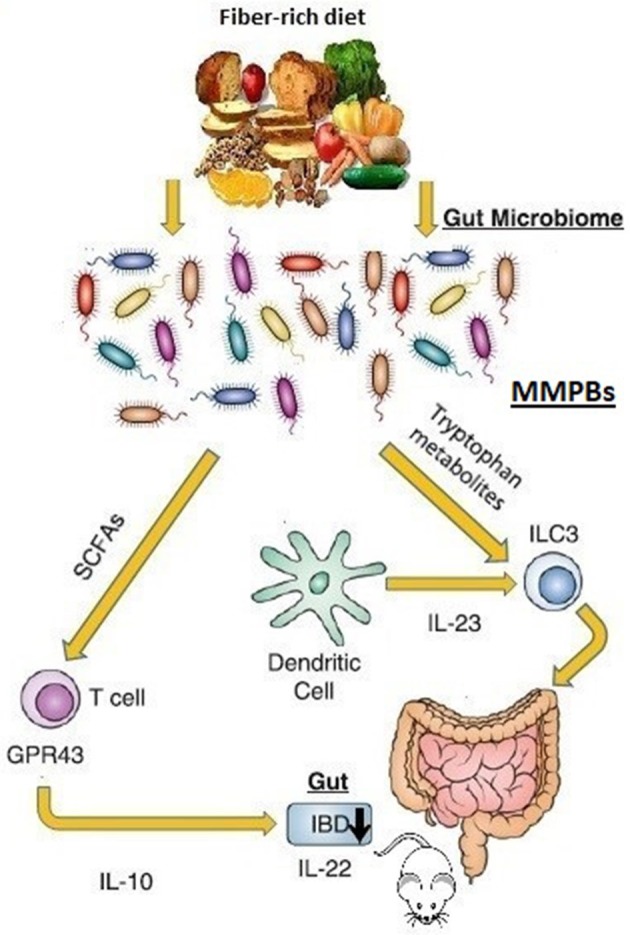
Microbiome-modulated post-biotics in IBD. The commensal bacteria produces an extremely diverse metabolite repertoire (microbiome-modulated post-biotics “MMPBs”) including SCFAs and tryptophan from dietary fiber fermentation. The MMBPs show immunomodulating effect (especially on the inflammation of the mice gastrointestinal tract), inducing the IL-22 secretion involved in pathogen resistance and mucosal protection. Altered levels of MMBPs have been also associated with IBD pathogenesis. Direct human evidence is lacking.

Another prominent paradigm of how the microbiota can affect the tissue-level immune maturation is the microbial metabolism of tryptophan. Trp metabolites have been identified in experimental colitis or IBD patients to have immunomodulatory activity (86). The aryl hydrocarbon receptor (AhR) is a cytoplasmic transcription factor found in several types of immune cells, as macrophages, DCs, IECs, B cells, and T cells (87). Kynurenine is a metabolic product derived from tryptophan that could be an endogenous AhR ligand (88). It is involved in the modulation of innate lymphoid cells expansion, intraepithelial lymphocytes, inflammatory, and immune reactions and contributes to the maintenance of normal mucosal function in the intestine (89). Diet-derived AhR ligands induce the IL-22 secretion (81, 90), which in turn favors the production of mucin and antimicrobial peptides in the gut, thus conferring pathogen resistance and mucosal protection (91). A division of commensal bacteria employs Trp as an energy source and produces the indole-3-aldeyde, which further activates AhR in ILCs, inducing the IL-22 secretion (Figure 2). This process affects both mucosal healing and the anti-microbial peptides repertoire including lipocalin-2, S100A8, and S100A9 in mice (92). Indole reduces the expression of inflammatory genes and up-regulates the expression of tight junction proteins (93) interacting with the AhR (94). In mice, the AhR activation, using tryptophan metabolites reduces dextran sulfate sodium-induced colitis (95).

The gut microflora of mice lacking caspase recruitment domain family member 9 (CARD9) was unable to metabolize Trp, the products of which would otherwise stimulate AhR. These mice models presented a reduced number of colonic IL-17A and IL-6 as well as fewer IL-22-producing innate lymphoid cells in the colon lamina propria (96). Conventionalization of germ free mice with the GM from CARD9 defective mice increased their susceptibility to develop colitis. Interestingly, gut inflammation in these mice was ameliorated with both AhR agonist supplementation and three *Lactobacillus* strains capable of metabolizing tryptophan. Furthermore, GM obtained from IBD patients were less able to produce AhR ligands (97). These results indicate that also Trp metabolites are bioactive mediators that modulate the crosstalk between GM ecosystem and the host immune response (98).

## Immunonutrition as a Novel IBD Therapeutic Approach?

Immunonutrition refers to the effects that specific dietary factors, as reported above, can have on different aspects of the immune system as well as on the microbiota. However, the role showed by these specific dietary factors in the pathophysiology of many metabolic disorders, specifically in promoting inflammation, raises the possibility that in IBD pathogenesis a heightened susceptibility to environmentally driven dysregulated intestinal immunity (i.e., that caused by a Western-style diet or by other factors of microbial dysbiosis) plays a prominent role. It was observed that the number of dominant SCFAs producing bacteria (i.e., *Faecalibacterium prausnitzii* and *Roseburia intestinalis*) decreased in IBD patients, consequently affecting the differentiation and expansion of Tregs as well as the growth of bowel epithelial cells (99). In this case, a counter-regulatory measure based on a dietary immunomodulation, clinically known as immunonutrition, could help to re-balance the inflamed bowel toward a neutral condition. Even if immunonutrition, to be adopted in the short-term perioperative course, is actually recommended (in recent surgical guidelines) to prepare oncologic patients for tumor removal (100), in IBD few studies on this topic are available in literature (101). In particular, the role of SCFAs and Trp post-biotics has been poorly evaluated as a novel therapeutic approach in CD or in UC, both in the perioperative period or in the long-term post-operative course. In experimental models, it was evaluated that GPR109a^−/−^ mice are at higher risk of developing colonic inflammation and cancer, and that niacin induces anti-inflammatory and anti-carcinogenic environment in the bowel via GPR109, demonstrating the therapeutic potential for this receptor (102). On the other hand, in individuals with mild to moderate UC in a Phase 2 randomized, double-blind, placebo-controlled clinical trial, GLPG0974, a GPR43-specific antagonist, even reducing the inflammatory response *in vitro*, did not change clinical outcomes of patients over a short period of time (103). Disagreeing data regarding the role of these receptors in either promotion or suppression of disease are still present, and how combinations or single specific SCFAs and Trp post-biotics contribute to beneficial or pathogenic effects in the host is still largely to be clarified (104).

Studies in which SCFAs were provided by different forms including oral administration and use of enemas were performed to investigate their various positive effects on IBD models in rats. In humans, although SCFAs or compounds (i.e., dietary fibers) that increase the availability of SCFAs, present beneficial *in vitro* effects in intestinal inflammation, the real life clinical positive effect is not consensual (105). In particular, it has been reported that SCFAs enemas increase mucosal generation, crypt length, and DNA content of the colonocytes, improving the UC symptoms in patients and rats injected with trinitrobenzene sulfonic acid (106). However, in UC patients in clinical remission daily administering 60 ml rectal enemas, containing 100 mM sodium butyrate (*n* = 17) or saline (*n* = 18) for a period of 20 days, butyrate enemas induced only minor effects on colonic inflammation and oxidative stress (107).

Furthermore, in IBD this dietetic approach could be useful in reducing the recurrence risk in CD patients and in preventing pouchitis after total proctocolectomy and ileo-pouch-anal anastomosis in UC patients.

Knowledge about nutritional intervention in CD patients is still extremely poor. For this reason, the most recent review about this topic report on the nutritional manipulation of GM and mucosal immune system is still only a promising prophylactic intervention against bowel inflammation (108). In fact, in addition to correcting malnutrition (factor connected with a better short-term post-operative outcome in CD) immunonutrition, enhancing immune function, may also induce CD remission or reduce the extent of resection needed by decreasing the inflammatory response.

In standard perioperative immunonutrition in oncologic patients, the key immune-modulating nutrients include ariginine, glutamine, nucleotides and omega-3 fatty acids either alone or in combination (100). In fact, surgical stress can cause an acute depletion of arginine, which both impairs T cell function and wound healing. This acute nutritional deficiency is potentially modifiable and has been the target of nutritional optimization around the surgery time. Nutritional supplements enriched with immunonutrients have recently been introduced into clinical practice for patients undergoing cancer surgery (109). Theoretically, the role of these immunonutrients administered in the perioperative period could be similar in oncologic patients in order to decrease the incidence of surgical complications, in particular infectious complications. As a supplement, the use of omega-3 fatty acids may also decrease the production of proinflammatory leukotrienes and prostaglandins by competing with omega-6 fatty acids in the eicosanoid pathway (110). This has been shown to reduce the steroid requirements in UC. In CD, where the C-reactive protein and erythrocyte sedimentation rate are elevated, omega-3 fatty acids have been shown to lower production of proinflammatory interferons and prostaglandins; however, it has not been shown to play a significant effect on disease activity and further studies on this are legitimate needed (111).

Preoperative preparation of patients with Crohn's disease is challenging and there are no specific guidelines regarding nutritional support. It has been shown that the optimization of the nutritional status before surgery (with either enteral or parenteral support) reduces post-operative intra-abdominal septic complications and the need for a temporary diverting stoma (112). More recently a retrospective, cohort study including all CD patients who underwent abdominal surgery showed that nutritional support can minimize post-operative complications in IBD patients with low albumin levels (113).

In addition, a recent study demonstrated that the microbial metabolite Urolithin A (UroA) (a major microbial metabolite derived from polyphenolics of berries and pomegranate fruits), and its analog UAS03, significantly enhance the gut barrier functionality and inhibit unwarranted inflammation. The oral treatment with UroA/UAS03 considerably mitigated systemic inflammation and colitis suggesting potential therapeutic applications for the protection from colonic diseases and the IBD treatment (114).

An alternative approach based on a dietary modulation targeting the immune system toward the GM post-biotics should be more widely evaluated in order to better refine the specific IBD pathogenesis and to decrease the incidence of short and long-term post-operative complications in both CD and UC.

## Conclusions

It is established that the gut colonization by the bacterial community is essential for a correct training of the immune system, in order to discriminate pathogens and commensals. Here we underline some examples by which microbial post-biotics could regulate host immune response in IBD. Currently, growing data documents immunomodulating effects of MMPSs in a wide range of immune cells (from innate to adaptive immunity), supporting a challenging frontier in the prevention and treatment of IBD. Examining the impact of bacterial metabolites on gut immunity, whether through direct effects on host immune cells or indirect effects on the GM, would provide us with critical information essential to promote innovative therapies.

Currently, the use of post-biotics has opened a new opportunity to search for and investigate the potential uses of microbiota-derived products as novel therapies for many inflammatory diseases. Nowadays, IBD therapies focus on the suppression of inflammation that characterizes IBD and the restoration of intestinal barriers. Immunonutrition is an underexploited and understudied topic of research that could provide a tractable approach employed in the future to reduce damaging intestinal inflammation in IBD.

## Author Contributions

ER and AA: conceptualization. ER, FG, and CF: investigation and writing. AA, FF, ER, and SS: review. ER: editing and visualization. ER and AA: supervision. FG, ER, FF, and AA: funding acquisition.

### Conflict of Interest

The authors declare that the research was conducted in the absence of any commercial or financial relationships that could be construed as a potential conflict of interest.
